# *N*-(4-Chloro­phen­yl)-5-(4,5-dihydro-1*H*-imidazol-2-yl)thieno[2,3-*b*]pyridin-4-amine

**DOI:** 10.1107/S160053681202658X

**Published:** 2012-06-20

**Authors:** Alice M. R. Bernardino, Luiz C. S. Pinheiro, Edward R. T. Tiekink, James L. Wardell, Solange M. S. V. Wardell

**Affiliations:** a Universidade Federal Fluminense, Instituto de Química, Departamento de Química Orgânica, Programa de Pós-Graduação em Química Orgânica, Campus do Valonguinho, CEP 24210-150 Niterói, RJ, Brazil; b Fundação Oswaldo Cruz, Instituto de Tecnologia em Fármacos, Departamento de Síntese Orgânica, Manguinhos, CEP 21041-250, Rio de Janeiro, RJ, Brazil; c Department of Chemistry, University of Malaya, 50603 Kuala Lumpur, Malaysia; d Centro de Desenvolvimento Tecnológico em Saúde (CDTS), Fundação Oswaldo Cruz (FIOCRUZ), Casa Amarela, Campus de Manguinhos, Av. Brasil 4365, 21040-900 Rio de Janeiro, RJ, Brazil; e CHEMSOL, 1 Harcourt Road, Aberdeen AB15 5NY, Scotland

## Abstract

In the title compound, C_16_H_13_ClN_4_S, the thienopyridine fused-ring system is nearly planar (r.m.s. deviation = 0.0333 Å) and forms a dihedral angle of 4.4 (3)° with the attached dihydro­imidazole ring (r.m.s. deviation = 0.0429 Å) allowing for the formation of an intra­molecular (exocyclic amine)N—H⋯N(imine) hydrogen bond. The benzene rings of the disordered (50:50) –N(H)—C_6_H_4_Cl residue form dihedral angles of 59.1 (3) and 50.59 (15)° with the fused ring system. In the crystal, (imidazole amine)N—H⋯N(pyridine) hydrogen bonds lead to a supra­molecular helical chain along the *b* axis. The chains assemble into layers (*ab* plane) with inter-digitation of the chloro­benzene rings which results in weak C—H⋯Cl inter­actions in the *c*-axis direction.

## Related literature

For the synthesis and biological activity of thienopyridine derivatives, see: Kaigorodova *et al.* (2000 ▶); Moloney (2001 ▶); Bernardino *et al.* (2004 ▶, 2006 ▶); Leal *et al.* (2008 ▶); Pinheiro *et al.* (2008*a* ▶); El-Kashef *et al.* (2010 ▶); Testa *et al.* (2010 ▶); Panchamukhi *et al.* (2011 ▶). For the anti-leishmanial activity of 5-(4,5-dihydro-1*H*-imidazol-2-yl)-4-(aryl­amino)­thieno[2,3-*b*]pyridine, see: Pinheiro *et al.* (2012 ▶).
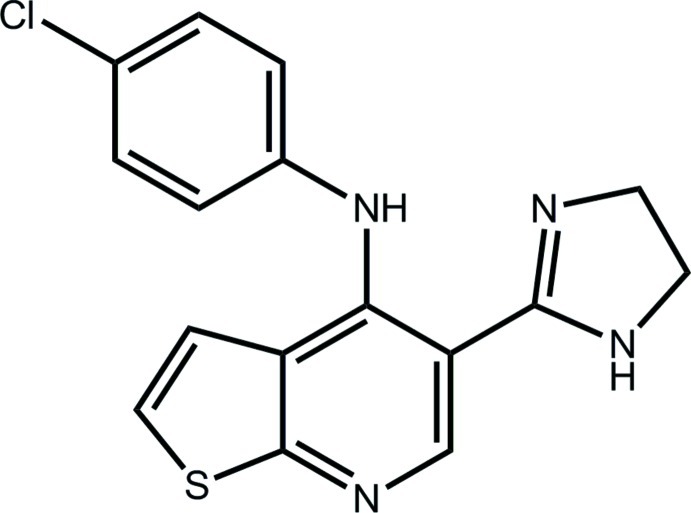


## Experimental

### 

#### Crystal data

C_16_H_13_ClN_4_S
*M* *_r_* = 328.81
Monoclinic, 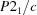

*a* = 17.784 (3) Å
*b* = 6.2264 (4) Å
*c* = 13.6226 (18) Å
β = 102.700 (4)°
*V* = 1471.5 (3) Å^3^
*Z* = 4
Mo *K*α radiation
μ = 0.40 mm^−1^
*T* = 120 K
0.25 × 0.15 × 0.03 mm

#### Data collection

Bruker–Nonius Roper CCD camera on κ-goniostat diffractometer
Absorption correction: multi-scan (*SADABS*; Sheldrick, 2007 ▶) *T*_min_ = 0.639, *T*_max_ = 1.000
9375 measured reflections
2591 independent reflections
1106 reflections with *I* > 2σ(*I*)
*R*_int_ = 0.138

#### Refinement

*R*[*F* ^2^ > 2σ(*F* ^2^)] = 0.079
*wR*(*F* ^2^) = 0.217
*S* = 0.99
2591 reflections
191 parameters
2 restraints
H atoms treated by a mixture of independent and constrained refinement
Δρ_max_ = 0.22 e Å^−3^
Δρ_min_ = −0.42 e Å^−3^

### 

Data collection: *COLLECT* (Hooft, 1998 ▶); cell refinement: *DENZO* (Otwinowski & Minor, 1997 ▶) and *COLLECT*; data reduction: *DENZO* and *COLLECT*; program(s) used to solve structure: *SHELXS97* (Sheldrick, 2008 ▶); program(s) used to refine structure: *SHELXL97* (Sheldrick, 2008 ▶); molecular graphics: *ORTEP-3 for Windows*(Farrugia, 1997 ▶) and *DIAMOND* (Brandenburg, 2006 ▶); software used to prepare material for publication: *publCIF* (Westrip, 2010 ▶).

## Supplementary Material

Crystal structure: contains datablock(s) global, I. DOI: 10.1107/S160053681202658X/xu5563sup1.cif

Structure factors: contains datablock(s) I. DOI: 10.1107/S160053681202658X/xu5563Isup2.hkl

Supplementary material file. DOI: 10.1107/S160053681202658X/xu5563Isup3.cml

Additional supplementary materials:  crystallographic information; 3D view; checkCIF report

## Figures and Tables

**Table 1:** Hydrogen-bond geometry (Å, °)

*D*—H⋯*A*	*D*—H	H⋯*A*	*D*⋯*A*	*D*—H⋯*A*
N2—H2n⋯N3	0.88 (10)	1.87 (11)	2.578 (18)	136 (10)
N2′—H2n’⋯N3	0.88 (10)	2.04 (11)	2.740 (15)	135 (7)
N4—H4n⋯N1^i^	0.88 (3)	2.10 (3)	2.956 (8)	167 (5)
C6—H6⋯Cl1′^ii^	0.95	2.74	3.559 (10)	146

Symmetry codes: (i) 
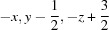
; (ii) 
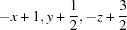
.

**Fig. 1.**
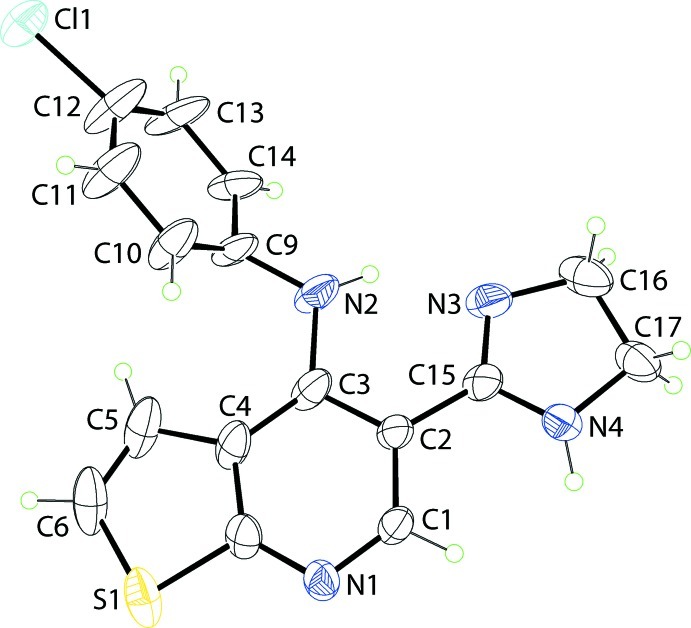
The molecular structure of (I) showing the atom-labelling scheme and displacement ellipsoids at the 35% probability level. Only one orientation of the disordered —N(H)—C6H4Cl residue is shown.

**Fig. 2.**
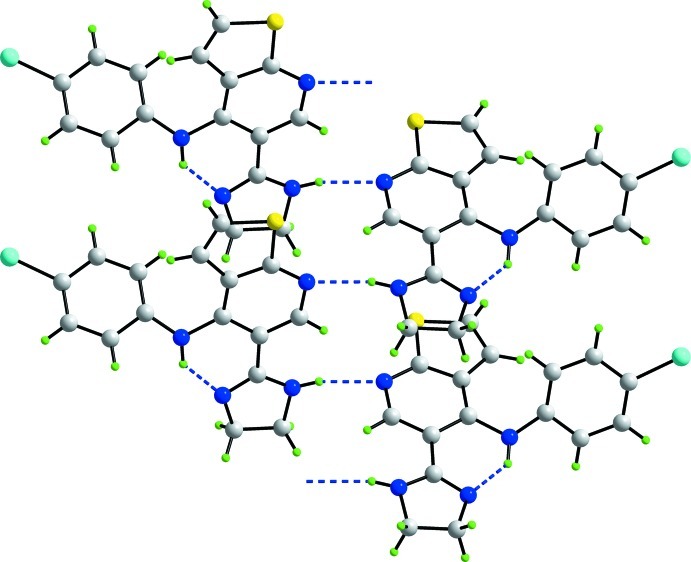
A view of the supramolecular helical chain propagated along [010] in (I) showing intra- and inter-molecular N—H···N (blue dashed lines) hydrogen bonds.

**Fig. 3.**
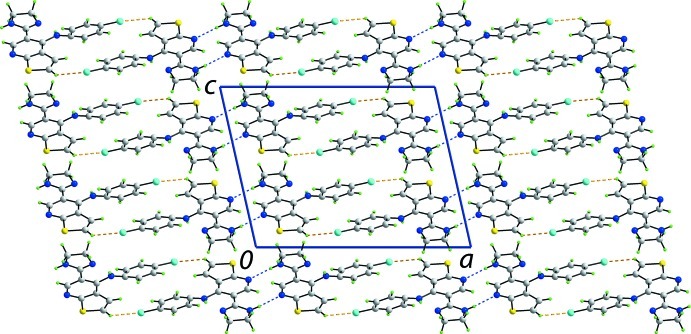
A view in projection down the b axis of the unit-cell contents for (I). The N—H···N and C—H···Cl interactions are shown as blue and orange dashed lines, respectively.

## supplementary crystallographic information

### Comment

Thienopyridine derivatives have been synthesized by a variety of routes (Kaigorodova *et al.*, 2000; Bernardino *et al.*, 2006; Pinheiro *et al.*, 2008; El-Kashef *et al.*, 2010; Testa *et al.*, 2010). A primary motivation for the preparation of these compounds is their biological activity, *viz*. anti-viral (Bernardino *et al.*, 2004), anti-inflammatory (Moloney, 2001), anti-bacterial (Leal *et al.*, 2008; Pinheiro *et al.*, 2008; Panchamukhi *et al.*, 2011) and anti-parasitic (Bernardino *et al.*, 2006). Recently, the anti-leishmanial activity of a family of 5-(4,5-dihydro-1*H*-imidazol-2-yl)-4-(arylamino)thieno[2,3-*b*]pyridine derivatives was reported (Pinheiro *et al.*, 2012). We now wish to report the crystal structure determination of a related derivative, namely 5-(4,5-dihydro-1*H*-imidazol-2-yl)-4-(4'-chlorophenylamino)-thieno[2,3-*b*]pyridine, (I).

In (I), Fig. 1, the nine non-hydrogen atoms of the thienopyridine ring are planar, having a r.m.s. deviation = 0.0333 Å and maximum deviations of 0.051 (5) Å [for the C7 atom] and -0.038 (5) Å [C6]. The imidazolyl ring is approximately planar [r.m.s. deviation = 0.0429 Å] and is co-planar with the fused ring system forming a dihedral angle of 4.4 (3)°. The imine-N3 atom of the imidazolyl ring is orientated towards the exocyclic amine so that an intramolecular hydrogen bond is formed, Table 1. There are two orientations for the disordered —N(H)—C_6_H_4_Cl residue of equal weight. The benzene rings of this residue are approximately co-planar (dihedral angle = 8.7 (5)°) and slightly displaced from each other. The dihedral angles between each orientation and the fused ring system are 59.1 (3) and 50.59 (15)°.

The most prominent feature of the crystal packing is the formation of N—H···N hydrogen bonds between the imidazolyl-amine and the pyridyl-N atom which lead to supramolecular helical chains along the *b* axis, Fig. 2 and Table 1. These assemble into layers in the *ab* plane allowing for inter-digitation of the chlorobenzene rings which in turn, allows for the formation of weak C—H···Cl interactions, Table 1. For the illustrated orientation of disordered benzene ring, Fig. 3, the H6···Cl1 separation is 2.95 Å.

### Experimental

Following general procedures (Bernardino *et al.*, 2006; Pinheiro *et al.*, 2012), a solution of 4-(4'-chlorophenylamino)thieno[2,3-*b*]pyridine-5-carbonitrile (1.5 mmol) in ethylenediamine (5 ml) was cooled at 273 K, carbon disulfide (8 drops) was added and the reaction mixture heated at 373 for 24 h. The resulting mixture was cooled, treated with water and filtered to give a brown crystalline solid, which was collected and dried. The sample used in the structure determination was grown from CHCl_3_ solution. IR (KBr, cm^-1^): ν NH 3225, ν C═N 1591). ^1^H NMR (300 MHz, CDCl_3_, TMS, δ in p.p.m.) 7.09 (d, 6.0, 1H, H2); 6.50 (d, 6.0, 1H, H3); 8.51 (s, 1H, H6); 7.29 (d, 8.7, 2H, Ar—H); 7.09 (d, 8.7, 2H, Ar—H); 3.83 (s, 4H, CH_2_). ^13^C NMR (75 MHz, DMSO-d6, TMS, δ in p.p.m.) 164.4; 164.1; 146.9; 146.6; 140.0; 129.3; 128.8; 125.4; 123.6; 121.3; 119.4; 105.4. ESI-(+)-MS [*M*+H]+ - 329.051 (100).

### Refinement

The C-bound H atoms were geometrically placed (C—H = 0.95–0.99 Å) and refined as riding with *U*_iso_(H) = 1.2*U*_eq_(C). The N-bound H atoms were located from a difference map and refined with a distance restraint of N—H = 0.88±0.01 Å, and with *U*_iso_(H) = 1.2*U*_eq_(carrier atom). The —N(H)—C_6_H_4_Cl residue was disordered over two position. From anisotropic refinement (equivalent pairs of atoms were tied, and C_6_ rings were idealized) the orientations were equal and so in the final refinement the site occupancies factors were fixed at 0.5. Several reflections, *i.e*. (1 0 0), (2 0 0), (0 0 2) and (-1 0 2), were affected by the beam-stop and were omitted from the final refinement.

### Crystal data

**Table:**

C_16_H_13_ClN_4_S	*F*(000) = 680
*M**_r_* = 328.81	*D*_x_ = 1.484 Mg m^−^^3^
Monoclinic, *P*2_1_/*c*	Mo *K*α radiation, λ = 0.71073 Å
Hall symbol: -P 2ybc	Cell parameters from 5430 reflections
*a* = 17.784 (3) Å	θ = 1.0–26.4°
*b* = 6.2264 (4) Å	µ = 0.40 mm^−^^1^
*c* = 13.6226 (18) Å	*T* = 120 K
β = 102.700 (4)°	Plate, colourless
*V* = 1471.5 (3) Å^3^	0.25 × 0.15 × 0.03 mm
*Z* = 4	

### Data collection

**Table:**

Bruker–Nonius Roper CCD camera on κ-goniostat diffractometer	2591 independent reflections
Radiation source: Bruker-Nonius FR591 rotating anode	1106 reflections with *I* > 2σ(*I*)
Graphite monochromator	*R*_int_ = 0.138
Detector resolution: 9.091 pixels mm^-1^	θ_max_ = 25.0°, θ_min_ = 3.4°
φ & ω scans	*h* = −21→21
Absorption correction: multi-scan (*SADABS*; Sheldrick, 2007)	*k* = −7→7
*T*_min_ = 0.639, *T*_max_ = 1.000	*l* = −16→16
9375 measured reflections	

### Refinement

**Table:**

Refinement on *F*^2^	Primary atom site location: structure-invariant direct methods
Least-squares matrix: full	Secondary atom site location: difference Fourier map
*R*[*F*^2^ > 2σ(*F*^2^)] = 0.079	Hydrogen site location: inferred from neighbouring sites
*wR*(*F*^2^) = 0.217	H atoms treated by a mixture of independent and constrained refinement
*S* = 0.99	*w* = 1/[σ^2^(*F*_o_^2^) + (0.088*P*)^2^ + 1.3236*P*] where *P* = (*F*_o_^2^ + 2*F*_c_^2^)/3
2591 reflections	(Δ/σ)_max_ < 0.001
191 parameters	Δρ_max_ = 0.22 e Å^−^^3^
2 restraints	Δρ_min_ = −0.42 e Å^−^^3^

### Special details

**Table:**

Geometry. All s.u.'s (except the s.u. in the dihedral angle between two l.s. planes) are estimated using the full covariance matrix. The cell s.u.'s are taken into account individually in the estimation of s.u.'s in distances, angles and torsion angles; correlations between s.u.'s in cell parameters are only used when they are defined by crystal symmetry. An approximate (isotropic) treatment of cell s.u.'s is used for estimating s.u.'s involving l.s. planes.
Refinement. Refinement of *F*^2^ against ALL reflections. The weighted *R*-factor *wR* and goodness of fit *S* are based on *F*^2^, conventional *R*-factors *R* are based on *F*, with *F* set to zero for negative *F*^2^. The threshold expression of *F*^2^ > 2σ(*F*^2^) is used only for calculating *R*-factors(gt) *etc*. and is not relevant to the choice of reflections for refinement. *R*-factors based on *F*^2^ are statistically about twice as large as those based on *F*, and *R*- factors based on ALL data will be even larger.

### Fractional atomic coordinates and isotropic or equivalent isotropic displacement parameters (Å^2^)

**Table:**

	*x*	*y*	*z*	*U*_iso_*/*U*_eq_	Occ. (<1)
S1	0.12641 (12)	0.5151 (2)	0.60112 (15)	0.0763 (7)	
N1	0.0697 (3)	0.2115 (6)	0.7026 (4)	0.0512 (13)	
N3	0.2209 (3)	−0.3570 (7)	0.8955 (4)	0.0739 (18)	
N4	0.0959 (3)	−0.3191 (7)	0.8940 (4)	0.0549 (14)	
H4N	0.0473 (11)	−0.288 (8)	0.869 (4)	0.066*	
C1	0.0829 (3)	0.0361 (8)	0.7610 (4)	0.0462 (14)	
H1	0.0394	−0.0312	0.7777	0.055*	
C2	0.1545 (3)	−0.0563 (7)	0.7995 (4)	0.0464 (15)	
C3	0.2210 (3)	0.0395 (8)	0.7765 (5)	0.0601 (18)	
C4	0.2088 (4)	0.2238 (8)	0.7136 (5)	0.0592 (18)	
C5	0.2606 (4)	0.3478 (10)	0.6675 (6)	0.081 (2)	
H5	0.3144	0.3211	0.6777	0.097*	
C6	0.2236 (5)	0.5055 (10)	0.6084 (6)	0.087 (2)	
H6	0.2493	0.6035	0.5736	0.104*	
C7	0.1334 (4)	0.2970 (8)	0.6822 (5)	0.0526 (16)	
Cl1	0.6039 (3)	0.3366 (8)	0.9082 (4)	0.0739 (13)	0.50
N2	0.2952 (7)	−0.067 (3)	0.8204 (17)	0.074 (3)	0.50
H2N	0.292 (7)	−0.200 (8)	0.842 (9)	0.089*	0.50
C8	0.3602 (3)	0.0556 (17)	0.8170 (11)	0.066 (3)	0.50
C9	0.3724 (3)	0.2610 (16)	0.8574 (12)	0.083 (3)	0.50
H9	0.3297	0.3483	0.8629	0.099*	0.50
C10	0.4470 (3)	0.3387 (12)	0.8896 (11)	0.093 (3)	0.50
H10	0.4554	0.4790	0.9172	0.111*	0.50
C11	0.5095 (3)	0.2109 (10)	0.8815 (5)	0.100 (3)	0.50
C12	0.4973 (4)	0.0056 (14)	0.8411 (8)	0.095 (4)	0.50
H12	0.5400	−0.0817	0.8356	0.113*	0.50
C13	0.4227 (4)	−0.0721 (16)	0.8089 (10)	0.071 (4)	0.50
H13	0.4143	−0.2124	0.7813	0.085*	0.50
Cl1'	0.6109 (3)	0.2495 (8)	0.9508 (4)	0.0739 (13)	0.50
N2'	0.2871 (4)	−0.045 (2)	0.8004 (14)	0.074 (3)	0.50
H2N'	0.2828	−0.1058	0.8574	0.089*	0.50
C8'	0.3706 (3)	0.0220 (11)	0.8521 (6)	0.066 (3)	0.50
C9'	0.3835 (4)	0.2370 (11)	0.8780 (8)	0.083 (3)	0.50
H9'	0.3412	0.3287	0.8805	0.099*	0.50
C10'	0.4582 (4)	0.3178 (14)	0.9004 (9)	0.093 (3)	0.50
H10'	0.4670	0.4647	0.9181	0.111*	0.50
C11'	0.5200 (4)	0.1836 (17)	0.8968 (9)	0.100 (3)	0.50
C12'	0.5071 (3)	−0.0313 (16)	0.8709 (9)	0.095 (4)	0.50
H12'	0.5494	−0.1230	0.8685	0.113*	0.50
C13'	0.4325 (3)	−0.1121 (14)	0.8486 (7)	0.071 (4)	0.50
H13'	0.4237	−0.2590	0.8309	0.085*	0.50
C14	0.1587 (2)	−0.2514 (6)	0.8627 (3)	0.0494 (15)	
C15	0.2003 (2)	−0.5287 (6)	0.9594 (4)	0.079 (2)	
H15A	0.2272	−0.5067	1.0304	0.094*	
H15B	0.2153	−0.6706	0.9371	0.094*	
C16	0.1138 (3)	−0.5183 (6)	0.9492 (3)	0.0678 (19)	
H16A	0.0875	−0.6421	0.9108	0.081*	
H16B	0.1000	−0.5117	1.0157	0.081*	

### Atomic displacement parameters (Å^2^)

**Table:**

	*U*^11^	*U*^22^	*U*^33^	*U*^12^	*U*^13^	*U*^23^
S1	0.1145 (17)	0.0388 (9)	0.0898 (14)	0.0007 (9)	0.0533 (12)	0.0066 (8)
N1	0.051 (3)	0.038 (3)	0.068 (4)	0.004 (2)	0.020 (3)	0.007 (2)
N3	0.048 (3)	0.035 (3)	0.121 (5)	−0.001 (2)	−0.019 (3)	0.006 (3)
N4	0.051 (3)	0.048 (3)	0.060 (4)	0.000 (3)	0.000 (3)	0.011 (2)
C1	0.037 (4)	0.043 (3)	0.059 (4)	−0.007 (3)	0.012 (3)	−0.004 (3)
C2	0.037 (4)	0.032 (3)	0.066 (4)	−0.002 (2)	0.003 (3)	−0.002 (3)
C3	0.035 (4)	0.033 (3)	0.113 (6)	−0.002 (3)	0.018 (4)	−0.016 (3)
C4	0.055 (4)	0.031 (3)	0.100 (5)	−0.007 (3)	0.034 (4)	−0.013 (3)
C5	0.082 (5)	0.050 (4)	0.129 (7)	−0.024 (4)	0.061 (5)	−0.024 (4)
C6	0.129 (7)	0.041 (4)	0.113 (6)	−0.019 (4)	0.077 (5)	−0.011 (4)
C7	0.060 (4)	0.032 (3)	0.071 (5)	0.002 (3)	0.027 (3)	−0.005 (3)
Cl1	0.0423 (14)	0.087 (4)	0.094 (4)	−0.012 (2)	0.019 (2)	−0.012 (2)
N2	0.031 (4)	0.041 (4)	0.144 (8)	0.002 (3)	0.004 (4)	−0.005 (4)
C8	0.022 (4)	0.043 (4)	0.126 (11)	0.008 (3)	−0.004 (5)	−0.012 (5)
C9	0.036 (4)	0.048 (4)	0.166 (9)	−0.002 (3)	0.028 (5)	−0.035 (5)
C10	0.038 (5)	0.083 (5)	0.162 (8)	−0.011 (4)	0.029 (5)	−0.061 (5)
C11	0.034 (5)	0.112 (6)	0.154 (8)	−0.021 (5)	0.022 (5)	−0.075 (6)
C12	0.022 (4)	0.102 (7)	0.147 (11)	0.013 (4)	−0.009 (5)	−0.057 (7)
C13	0.040 (5)	0.059 (5)	0.097 (12)	0.020 (4)	−0.020 (6)	−0.024 (7)
Cl1'	0.0423 (14)	0.087 (4)	0.094 (4)	−0.012 (2)	0.019 (2)	−0.012 (2)
N2'	0.031 (4)	0.041 (4)	0.144 (8)	0.002 (3)	0.004 (4)	−0.005 (4)
C8'	0.022 (4)	0.043 (4)	0.126 (11)	0.008 (3)	−0.004 (5)	−0.012 (5)
C9'	0.036 (4)	0.048 (4)	0.166 (9)	−0.002 (3)	0.028 (5)	−0.035 (5)
C10'	0.038 (5)	0.083 (5)	0.162 (8)	−0.011 (4)	0.029 (5)	−0.061 (5)
C11'	0.034 (5)	0.112 (6)	0.154 (8)	−0.021 (5)	0.022 (5)	−0.075 (6)
C12'	0.022 (4)	0.102 (7)	0.147 (11)	0.013 (4)	−0.009 (5)	−0.057 (7)
C13'	0.040 (5)	0.059 (5)	0.097 (12)	0.020 (4)	−0.020 (6)	−0.024 (7)
C14	0.045 (4)	0.035 (3)	0.061 (4)	−0.009 (3)	−0.003 (3)	−0.011 (3)
C15	0.091 (6)	0.042 (4)	0.082 (5)	0.002 (3)	−0.026 (4)	−0.001 (3)
C16	0.077 (5)	0.044 (3)	0.068 (5)	−0.007 (3)	−0.015 (4)	0.010 (3)

### Geometric parameters (Å, º)

**Table:**

S1—C6	1.710 (8)	C9—C10	1.3900
S1—C7	1.738 (6)	C9—H9	0.9500
N1—C7	1.336 (7)	C10—C11	1.3900
N1—C1	1.341 (6)	C10—H10	0.9500
N3—C14	1.281 (6)	C11—C12	1.3900
N3—C15	1.474 (6)	C12—C13	1.3900
N4—C14	1.348 (6)	C12—H12	0.9500
N4—C16	1.449 (6)	C13—H13	0.9500
N4—H4N	0.877 (10)	Cl1'—C11'	1.674 (10)
C1—C2	1.390 (7)	N2'—C8'	1.556 (10)
C1—H1	0.9500	N2'—H2N'	0.88 (1)
C2—C3	1.421 (8)	C8'—C9'	1.3900
C2—C14	1.481 (6)	C8'—C13'	1.3900
C3—N2'	1.263 (12)	C9'—C10'	1.3900
C3—C4	1.420 (8)	C9'—H9'	0.9500
C3—N2	1.479 (16)	C10'—C11'	1.3900
C4—C7	1.391 (8)	C10'—H10'	0.9500
C4—C5	1.447 (8)	C11'—C12'	1.3900
C5—C6	1.347 (10)	C12'—C13'	1.3900
C5—H5	0.9500	C12'—H12'	0.9500
C6—H6	0.9500	C13'—H13'	0.9500
Cl1—C11	1.815 (9)	C15—C16	1.5151
N2—C8	1.394 (17)	C15—H15A	0.9900
N2—H2N	0.88 (1)	C15—H15B	0.9900
C8—C9	1.3900	C16—H16A	0.9900
C8—C13	1.3900	C16—H16B	0.9900
			
C6—S1—C7	90.3 (3)	C13—C12—C11	120.0
C7—N1—C1	113.7 (5)	C13—C12—H12	120.0
C14—N3—C15	105.7 (4)	C11—C12—H12	120.0
C14—N4—C16	109.2 (4)	C12—C13—C8	120.0
C14—N4—H4N	128 (4)	C12—C13—H13	120.0
C16—N4—H4N	119 (4)	C8—C13—H13	120.0
N1—C1—C2	126.0 (5)	C3—N2'—C8'	138.2 (13)
N1—C1—H1	117.0	C3—N2'—H2N	118 (6)
C2—C1—H1	117.0	C8'—N2'—H2N	92 (6)
C1—C2—C3	118.8 (5)	C3—N2'—H2N'	98.6
C1—C2—C14	118.9 (5)	C8'—N2'—H2N'	88.6
C3—C2—C14	122.3 (5)	C9'—C8'—C13'	120.0
N2'—C3—C4	120.3 (8)	C9'—C8'—N2'	117.5 (7)
N2'—C3—C2	122.7 (8)	C13'—C8'—N2'	120.4 (8)
C4—C3—C2	116.6 (5)	C8'—C9'—C10'	120.0
C4—C3—N2	127.6 (8)	C8'—C9'—H9'	120.0
C2—C3—N2	115.8 (8)	C10'—C9'—H9'	120.0
C7—C4—C3	117.4 (5)	C11'—C10'—C9'	120.0
C7—C4—C5	110.8 (6)	C11'—C10'—H10'	120.0
C3—C4—C5	131.6 (6)	C9'—C10'—H10'	120.0
C6—C5—C4	111.9 (7)	C10'—C11'—C12'	120.0
C6—C5—H5	124.0	C10'—C11'—Cl1'	122.2 (6)
C4—C5—H5	124.0	C12'—C11'—Cl1'	115.9 (6)
C5—C6—S1	114.6 (5)	C13'—C12'—C11'	120.0
C5—C6—H6	122.7	C13'—C12'—H12'	120.0
S1—C6—H6	122.7	C11'—C12'—H12'	120.0
N1—C7—C4	127.6 (5)	C12'—C13'—C8'	120.0
N1—C7—S1	119.9 (5)	C12'—C13'—H13'	120.0
C4—C7—S1	112.4 (4)	C8'—C13'—H13'	120.0
C8—N2—C3	114.5 (16)	N3—C14—N4	116.2 (4)
C8—N2—H2N	129 (8)	N3—C14—C2	123.6 (4)
C3—N2—H2N	116 (8)	N4—C14—C2	120.1 (4)
C3—N2—H2N'	93.2	N3—C15—C16	107.1 (2)
C9—C8—C13	120.0	N3—C15—H15A	110.3
C9—C8—N2	123.2 (15)	C16—C15—H15A	110.3
C13—C8—N2	111.9 (13)	N3—C15—H15B	110.3
C10—C9—C8	120.0	C16—C15—H15B	110.3
C10—C9—H9	120.0	H15A—C15—H15B	108.6
C8—C9—H9	120.0	N4—C16—C15	100.8 (2)
C11—C10—C9	120.0	N4—C16—H16A	111.6
C11—C10—H10	120.0	C15—C16—H16A	111.6
C9—C10—H10	120.0	N4—C16—H16B	111.6
C10—C11—C12	120.0	C15—C16—H16B	111.6
C10—C11—Cl1	117.2 (4)	H16A—C16—H16B	109.4
C12—C11—Cl1	122.2 (4)		
			
C7—N1—C1—C2	0.1 (8)	C8—C9—C10—C11	0.0
N1—C1—C2—C3	0.1 (8)	C9—C10—C11—C12	0.0
N1—C1—C2—C14	−179.7 (5)	C9—C10—C11—Cl1	171.4 (3)
C1—C2—C3—N2'	−173.2 (10)	C10—C11—C12—C13	0.0
C14—C2—C3—N2'	6.6 (13)	Cl1—C11—C12—C13	−170.9 (3)
C1—C2—C3—C4	−0.9 (8)	C11—C12—C13—C8	0.0
C14—C2—C3—C4	178.9 (5)	C9—C8—C13—C12	0.0
C1—C2—C3—N2	179.7 (10)	N2—C8—C13—C12	−156.1 (17)
C14—C2—C3—N2	−0.5 (11)	C4—C3—N2'—C8'	57 (3)
N2'—C3—C4—C7	174.0 (10)	C2—C3—N2'—C8'	−131.2 (19)
C2—C3—C4—C7	1.5 (8)	N2—C3—N2'—C8'	−87 (7)
N2—C3—C4—C7	−179.2 (11)	C3—N2'—C8'—C9'	−4 (3)
N2'—C3—C4—C5	−0.7 (13)	C3—N2'—C8'—C13'	−168.0 (18)
C2—C3—C4—C5	−173.2 (6)	C13'—C8'—C9'—C10'	0.0
N2—C3—C4—C5	6.1 (15)	N2'—C8'—C9'—C10'	−163.7 (10)
C7—C4—C5—C6	1.7 (8)	C8'—C9'—C10'—C11'	0.0
C3—C4—C5—C6	176.6 (6)	C9'—C10'—C11'—C12'	0.0
C4—C5—C6—S1	−1.2 (8)	C9'—C10'—C11'—Cl1'	−163.6 (6)
C7—S1—C6—C5	0.4 (5)	C10'—C11'—C12'—C13'	0.0
C1—N1—C7—C4	0.6 (8)	Cl1'—C11'—C12'—C13'	164.6 (6)
C1—N1—C7—S1	176.0 (4)	C11'—C12'—C13'—C8'	0.0
C3—C4—C7—N1	−1.5 (9)	C9'—C8'—C13'—C12'	0.0
C5—C4—C7—N1	174.3 (6)	N2'—C8'—C13'—C12'	163.2 (9)
C3—C4—C7—S1	−177.2 (4)	C15—N3—C14—N4	0.7 (6)
C5—C4—C7—S1	−1.4 (6)	C15—N3—C14—C2	176.6 (5)
C6—S1—C7—N1	−175.4 (5)	C16—N4—C14—N3	−7.2 (7)
C6—S1—C7—C4	0.6 (5)	C16—N4—C14—C2	176.7 (4)
N2'—C3—N2—C8	55 (5)	C1—C2—C14—N3	174.8 (5)
C4—C3—N2—C8	15 (3)	C3—C2—C14—N3	−5.0 (8)
C2—C3—N2—C8	−165.7 (17)	C1—C2—C14—N4	−9.4 (8)
C3—N2—C8—C9	55 (2)	C3—C2—C14—N4	170.8 (5)
C3—N2—C8—C13	−149.3 (11)	C14—N3—C15—C16	5.8 (4)
C13—C8—C9—C10	0.0	C14—N4—C16—C15	9.7 (5)
N2—C8—C9—C10	153.3 (17)	N3—C15—C16—N4	−9.2 (4)

### Hydrogen-bond geometry (Å, º)

**Table:**

*D*—H···*A*	*D*—H	H···*A*	*D*···*A*	*D*—H···*A*
N2—H2n···N3	0.88 (10)	1.87 (11)	2.578 (18)	136 (10)
N2′—H2n'···N3	0.88 (10)	2.04 (11)	2.740 (15)	135 (7)
N4—H4n···N1^i^	0.88 (3)	2.10 (3)	2.956 (8)	167 (5)
C6—H6···Cl1′^ii^	0.95	2.74	3.559 (10)	146

Symmetry codes: (i) −*x*, *y*−1/2, −*z*+3/2; (ii) −*x*+1, *y*+1/2, −*z*+3/2.
